# Medical Students

**Published:** 1881-12

**Authors:** 


### SELECTIONS.

### MEDICAL STUDENTS.

The following extract from an editorial in *The American Specialist* should receive the careful consideration of all students of dentistry as well as medicine:

It seems to us appropriate to address a few words of advice to the young gentlemen who will this fall commence the study of the greatest of professions. Of their own free wills they have chosen a pursuit full of the gravest responsibilities. So great and so serious, indeed, are these responsibilities, that were they properly comprehended beforehand, but few would have the courage to enter upon the study of this profession. Yet such should not be the case. No matter what responsibilities may be placed upon a man in this world, if he meets them to the best of his ability he has fully performed his duty, and no one can do more. But in order that he may meet the responsibilities of the future and do his best, it is absolutely essential that he should properly prepare himself. To prepare himself properly it is essential that he should go about his work in a systematic manner. The time passed in college, the period occupied in listening to lectures, reading text books, and performing the clinical work pertaining to a college education, is to the physicians after education what the foundation of a house is to the superstructure. The professors of a medical college are the architects of this foundation. Their experienced minds have drawn plans, which, if accurately followed, will cause the medical mental foundation of the students to be securely and firmly laid, capable of sustaining any amount of accumulation of knowledge in after years. Should the architect of a building prepare his plans with the greatest good judgment and care, and the masons perform their work carelessly and imperfectly, the resultant foundation will be so weak and so insecure that it will be impossible to erect upon it a massive and substantial building, even though the best of material may be at hand and the plans for the building may be perfect. The foundation being imperfectly prepared, will not be strong and thorough enough to support the proposed building, and the only way out of the dilemma will be to undo what has been poorly done, and starting again at the beginning, to build a substantial foundation, by which much valuable time will be wasted. The case is identical with the medical student. If, during his college days, he prepares a sound foundation, he will possess the mental groundwork upon which, in after years, he can erect a *massive mint* of medical knowledge, a mint in which he can coin not only reputation of the highest order, but money as well. If he has not this thorough intimacy with the principles of his profession when he leaves college, one of two things will inevitably result. 1st. He will always remain in the ranks, never rising to the dignity of a commissioned officer in his profession, because, no matter how brilliant or able he may be, no matter how hard he may work, no matter how great may be the opportunities offered to him, he will forever after be confronted and interfered with by the poor foundation he has laid, by the want of intimate knowledge of the fundamental doctrines and principles of his profession. For instance, suppose you neglect the study of anatomy. Your comprehension of surgery and your ability in physical diagnosis will ever after be limited, since for success in either an intimate knowledge of anatomy is necessary. 2d. After some years have passed and the wild oats of youth, sown thoughtlessly during your college days, have brought you a large crop of experience, you commence to think and reflect; from a wild boy, you have become transformed into a thoughtful man. This contemplation soon makes painfully evident to you the dismal fact that you are not a thorough physician. You will realize that your knowledge of the fundamental medical principles is very incomplete, and if ambitious, the painful truth dawns upon you, that, owing to your own foolishness, owing to your wasted opportunities, you are doomed to occupy a very ordinary position in your profession, while the position of a Hunter, of a Gross, an Agnew, or a Pepper, is forever denied to you. You finally accept what you consider to be the inevitable, and settle down to the drudgery of an ordinary mediocre routine doctor, and after passing many years of labor, you die without having achieved either fame or fortune. This will occur, no matter how able you may be naturally. This is the history in brief of the very large majority of physicians who neglect to thoroughly prepare themselves, when at college, for the future study and investigation of their lives. This is, no doubt, in part due to the unfortunate fact that young men of any age can commence the study of medicine, and that the majority of medical students have not reached the age of reason. Bible commentators tell us that the instance of the repentance of the dying thief on the Cross has been given in order to show us that death-bed repentance is possible, thus preventing the lifelong sinner from despairing of the possibility of forgiveness and dying in his sins. But they also tell us that this is the only instance of eleventh-hour repentance and forgiveness recorded in the Bible, from which they infer that while *possible,* such repentance is *very, very improbable.* A parallel case exists in medical history. While what I have pictured to you will surely be the experience of the large majority of physicians who, as students, have neglected to thoroughly master the first principles of their profession, yet we have some exceptions to this dismal rule, which serve as beacons of hope and encouragement to the thoughtful man who has been a wild and thoughtless youth. The brightest of these hopeful lights is found in the life of John Hunter, whom Professor Gross styles the "*Father of Scientific Surgery.*" As a boy and a young man, he was, though not dissipated, yet careless, wild, and thoughtless. Study was irksome to him. He was lazy. Reaching mature years, he realized the errors of his youth, and by dint of constant and herculean labor he rose rapidly in the ranks of his profession, and finally passed away, leaving behind him a name and a record which will, to the end of time, rank him among the greatest men who have lived. Such cases, I say, are rare, very rare, and should serve, not so much as a guide for all, but as an incentive to those who have foolishly wasted their early opportunities and realize their mistake when they come to mental maturity. Again, such a recovery of lost opportunities can be effected in only one way, namely, by commencing all over again, as in the case of the imperfect foundation for the house, and such a course must surely entail the loss or the consumption of much time, which fact alone will prevent all, except such as are endowed with remarkable moral courage and ambition, from adopting it.

After attending clinics, go home and carefully study all about the various cases that have been presented to you, and examine your room-mate upon them, and let him do the same to you. This private quiz will cost less and benefit you more than the public ones. Never leave a subject until you have committed to memory and thoroughly digested all that your text-books can tell you about it. Take advantage of all the opportunities for clinical instruction which your college course affords you. Combine the theoretical knowledge derived from careful study of your text-books with the clinical knowledge.

If you attend to my few suggestions, and follow the course which your professors have mapped out for you, you will, at the termination of your collegiate studies, leave your alma mater, thoroughly educated physicians, capable of standing alone and of pursuing your studies and investigations independently, and of building on a sound foundation; if you do not, you will only be half a doctor, and an everlasting drag on your noble profession.

